# Protecting Orthopaedic Implants from Infection: Antimicrobial Peptide Mel4 Is Non-Toxic to Bone Cells and Reduces Bacterial Colonisation When Bound to Plasma Ion-Implanted 3D-Printed PAEK Polymers

**DOI:** 10.3390/cells13080656

**Published:** 2024-04-09

**Authors:** Hedi Verena Kruse, Sudip Chakraborty, Renxun Chen, Naresh Kumar, Muhammad Yasir, William T. Lewin, Natalka Suchowerska, Mark D. P. Willcox, David R. McKenzie

**Affiliations:** 1 Arto Hardy Family Biomedical Innovation Hub, Chris O’Brien Lifehouse, Missenden Road, Camperdown, Sydney, NSW 2050, Australia; will.lewin@lh.org.au; 2 School of Physics, The University of Sydney, Sydney, NSW 2006, Australia; natalka.suchowerska@sydney.edu.au; 3 Sarcoma and Surgical Research Centre, Chris O’Brien Lifehouse, Missenden Road, Camperdown, Sydney, NSW 2050, Australia; 4 School of Chemistry, University of New South Wales, Sydney, NSW 2052, Australiar.chen@unsw.edu.au (R.C.); n.kumar@unsw.edu.au (N.K.); 5 School of Optometry and Vision Science, University of New South Wales, Sydney, NSW 2052, Australia; m.yasir@unsw.edu.au (M.Y.); m.willcox@unsw.edu.au (M.D.P.W.); 6 School of Medical Sciences, The University of Sydney, Sydney, NSW 2006, Australia

**Keywords:** antimicrobial peptide, 3D printing, plasma immersion ion implantation, polyether ether ketone, polyether ketone, biofilm, infection prevention, orthopaedic implant, selective laser sintering, filament deposition modelling

## Abstract

Even with the best infection control protocols in place, the risk of a hospital-acquired infection of the surface of an implanted device remains significant. A bacterial biofilm can form and has the potential to escape the host immune system and develop resistance to conventional antibiotics, ultimately causing the implant to fail, seriously impacting patient well-being. Here, we demonstrate a 4 log reduction in the infection rate by the common pathogen *S. aureus* of 3D-printed polyaryl ether ketone (PAEK) polymeric surfaces by covalently binding the antimicrobial peptide Mel4 to the surface using plasma immersion ion implantation (PIII) treatment. The surfaces with added texture created by 3D-printed processes such as fused deposition-modelled polyether ether ketone (PEEK) and selective laser-sintered polyether ketone (PEK) can be equally well protected as conventionally manufactured materials. Unbound Mel4 in solution at relevant concentrations is non-cytotoxic to osteoblastic cell line Saos-2. Mel4 in combination with PIII aids Saos-2 cells to attach to the surface, increasing the adhesion by 88% compared to untreated materials without Mel4. A reduction in mineralisation on the Mel4-containing surfaces relative to surfaces without peptide was found, attributed to the acellular portion of mineral deposition.

## 1. Introduction

Bone implants are required to provide mechanical support and to act as a scaffold for the regrowth of bone after a critical defect is created by surgery following disease or trauma. Immediately after insertion of the implant, there is an elevated risk of infection at the site [1]. Infections impede the bone healing process and can jeopardise the success of the implant. One of the most serious complications of infection is biofilm formation leading to immune system evasion on the implant surface which is difficult to control with conventional antibiotics. Strategies to reduce the risk are currently based on controlled release of antimicrobial agents into the tissue around the implant from a coating that remains active for sufficient time [2]. An alternative approach to the prevention of biofilm formation is by attaching antimicrobial agents directly to the surface at risk. Strong tethering in the form of covalent linkage on the surface has advantages over controlled release into the surrounding tissues, not the least of which is that the antimicrobial action is restricted to the site and is therefore less likely to create unwanted toxicity elsewhere in the body. Antimicrobial peptides are among the most successful of the antimicrobial agents that are currently under study and development [3]. Peptides have an advantage over other antimicrobials in that they are broad spectrum and are less likely to develop resistant populations of the infectious agents [4,5]. The development of resistance to peptides is minimised by the rapid killing kinetics and the multimodality of the mechanism of the antimicrobial action, in comparison with conventional antibiotics [6,7].

Titanium is a widely adopted material for use in orthopaedic implants with a considerable track record of good integration with the body and low toxicity. However, it has significant drawbacks for patients, especially those with bone cancer, as it brings challenges for medical imaging and radiation therapy. In addition, titanium causes stress shielding due to its high mechanical stiffness that may induce osteolysis around the implant and lead to loosening in the long term. These problems may be overcome with the use of high-strength polymers in the poly (aryl-ether-ketone) (PAEK) family such as polyether ether ketone (PEEK) and polyether ketone (PEK), which both possess bone-like density, stiffness, and radiolucency. The emergence of 3D printing technologies for these polymers has changed the landscape of orthopaedic implants to a considerable extent, so that currently these materials present an alternative to metallic materials such as titanium and its alloys. PAEK materials are readily fabricated by the 3D printing processes known as fused deposition modelling (FDM) and selective laser sintering (SLS). The FDM process developed for PEEK operates at high temperature and is able to produce strong constructs from a heated nozzle fed by a filament of the PEEK material. The selective laser sintering process produces objects from a powder precursor, in our case PEK, that is supplied to the build volume in layers which are subjected to sintering by a laser beam. SLS-printed objects have considerably rougher surfaces than FDM-printed ones due to the presence of powder grains that protrude from the median plane, creating cavities and consequently surface porosity. The protection of such rough surfaces from microbial infection presents a serious challenge, owing to the greater surface area and the potential for entrapment of microbes within cavities.

Without prior treatment, neither PEEK nor PEK surfaces are especially receptive to biological engagement, having a water contact angle that is borderline hydrophobic. However, the surface treatment method known as plasma immersion ion implantation (PIII) has a track record of success in improving biocompatibility of polymers by reducing the water contact angle and introducing strong binding sites for attaching biomolecules [8,9]. PIII is distinguished from conventional plasma treatments by the application of an electric potential difference, usually of more than one kV, to extract ions from the plasma and accelerate them into the surface of the polymer [10]. Strong evidence is available [11] that for carbon backbone polymers, persistent carbon-centred radicals are created in a reservoir below the surface that remains active for times of the order of months [12,13]. After diffusion to the surface [14], these radicals are able to initiate chemical bonds that can form links between the surface and biomolecules physically adsorbed on the surface. A detailed study of the reaction mechanism that links the amino acid cysteine to the surface of a PIII-treated plasma polymer [15] has revealed using XPS that the sulphur atom of the cysteine changes its chemical bonding state as a result of interaction with the radical rich PIII-treated surface. After PIII treatment, FDM-printed PEEK implants demonstrated strong osseointegration with sheep bone in an in vivo trial [16]. Micro-CT analysis and fixation strength testing using a torque test confirmed that osseointegration had occurred [16,17]. Given its chemical similarity to PEEK, it is expected that PEK should behave in a similar fashion in its response to the PIII process.

The literature on the use of antimicrobial peptides on dental implants has been reviewed [18]. Two main methods have been reported for the linking of antimicrobial peptides on the surface of PAEK polymers, 1-ethyl-3-(-3-dimethylaminopropyl) carbodiimide (EDC) chemistry [19] and “click” chemistry [20]. EDC chemistry has been used to immobilise Mel4 on glass surfaces, the bond strength has been tested and is consistent with covalency [21]. However, the use of chemical linkers requires additional steps compared to the use of PIII which only requires incubation with the molecule to be attached in solution. In addition, the presence of potentially harmful residues may cause concern for human implantation. As an example, antimicrobial peptide KR-12 has been applied to a PEEK surface using “click” chemistry; however, the results show only a reduction in bacterial adhesion by less than one log [22]. Covalent binding provides an important advantage since rapid removal of the peptides by other biomolecules that have a higher affinity for the surface is likely and is prevented by treating the surface with PIII [23]. The surface treatment with PIII is successful in reducing the contact angle and simultaneously enabling covalent binding of a wide range of biomolecules to carbon-backbone polymers. Examples of biomolecules with demonstrated covalent binding include catalase and poly-L-lysine bound to polyethylene [24], bone morphogenic protein and tropoelastin to PEEK [25], small-interfering RNAs to polystyrene microparticles [26], and melamine to polyvinyl chloride [27]. 

Before an infection prevention process using PIII treated PEEK or PEK can be adopted clinically, it is necessary to confirm the efficacy of antimicrobial activity of the bound antimicrobial peptide as well as the effect of the peptide on the biocompatibility of the material towards human tissue. 

In this work, we use the antimicrobial peptide Mel4 which has been developed and tested in human trials of contact lenses as an effective broad-spectrum antimicrobial agent [28,29] and in preclinical trials in rabbits with Mel4-coated titanium plates [30]. Mel4 shows antimicrobial activity against antibiotic-resistant strains of *Staphylococcus aureus* and *Pseudomonas aeruginosa*, as well as being active against fungi [31]. Previous work has shown that Mel4 retains its activity while attached on a surface [32,33]. Mel4 is a small cationic peptide with the amino acid sequence KNKRKRRRRRRGGRRRR [34]. In the case of Gram-negative bacteria, Mel4 acts on the outer lipopolysaccharide membrane and then acts on their cytoplasmic membrane, followed by release of cytoplasmic contents and lysis of the cells [7]. In the case of the Gram-positive bacterium *S. aureus*, Mel4 binds to lipoteichoic acid, releasing autolysins that can cleave the cell wall as well as disrupting the cytoplasmic membrane [6]. When covalently bound, Mel4 acts by similar mechanisms as it does in solution, albeit more slowly [21,33].

Since the 3D manufacturing process creates various surface topographies and roughness levels which may be a significant factor for the vulnerability of the surfaces to infection, we tested a commercially manufactured PEEK in the form of semi-crystalline sheet, an FDM-printed PEEK surface and an SLS-printed PEK surface. These three forms of the PAEK polymers are selected as application-ready forms of PAEK that could benefit from antimicrobial protection.

We used three established in vitro models to assess the use of Mel4 bound to PAEK polymers as 3D-printable antimicrobial orthopaedic implant materials. Firstly, water contact angle measurements were carried out [35]. Secondly, the antimicrobial effect of Mel4 bound to PAEK materials was investigated by a biofilm formation challenge test with *S. aureus* [21]. Finally, as a model to predict osseointegration behaviour of the Mel4 bound to PAEK in vivo, adhesion, proliferation, and mineralisation of the Saos-2 osteosarcoma cell line cultured on the substrates was assessed [36,37].

## 2. Materials and Methods

### 2.1. Sample Preparation

All specimens were cut with a 6 mm hole punch from sheets that were either as purchased or in-house manufactured by 3D printing. PEEK sheet specimens were cut from semicrystalline PEEK film with a 250 µm thickness (Aptiv sheet^®^, Victrex manufacturing Ltd., Thornton, Lancashire, United Kingdom). The sheets have a very smooth, optically reflective surface finish. FDM PEEK discs were cut from sheets prepared on an AON M.2 3D printer (AON3D, Montreal, QC, Canada) with ThermaX^TM^ PEEK filament (3DXTech, Grand Rapids, MI, USA, batch 49-122619-06JV). The average thickness of the sheets was 210 µm. The surface finish was smooth on the print bed (polyimide tape)-facing side; the top side shows tracks left by the 0.2 mm diameter printing nozzle. The FDM printing parameters are listed in Table 1. SLS PEK specimens were cut from SLS-printed PEK sheets that were prepared by selective laser sintering (SLS) using an EOS P800 system (EOS GmbH, Krailling, Germany); the printing parameters are listed in Table 2. The SLS process was interrupted after the sheets were laser sintered to prevent recoating with fresh powder, resulting in thinner sheets with one smooth face. The average thickness of the sheets was 290 µm. One face of the SLS PEK sheets showed a very rough surface, typical for SLS prints due to powder grains with a mean particle diameter of 60 µm. To remove un-sintered, loose particles after SLS printing, the sheets were dry ice-blasted with a ColdJet MicroClean (ColdJet, Loveland, OH, USA) at 3 bar gauge pressure, a feed rate of 0.10 kg/min, and a gentle fragmenting nozzle.

### 2.2. PIII Treatment 

The plasma immersion ion implantation process was carried out in a dielectric barrier discharge system consisting of a borosilicate flask with an external electrode made from copper foil as described previously [38]. The samples were placed in the base of the flask, the flask was evacuated to a base pressure of 2 × 10^−4^ mTorr, and nitrogen gas admitted to a pressure of 350 mtorr (low) or 700 mTorr (high). High-voltage pulses were applied to the electrode using an ANSTO custom-built power supply using 20 µs pulses of 1000 Hz repetition rate at a negative potential difference of −10 kV with a rise time of 500 ns between the copper external electrode and the metal vacuum chamber at earth potential to which the flask is attached. Plasma discharge was generated for a total time of 16 min at low pressure and 4 min at high pressure. The samples have been turned over in middle of the treatment to ensure even treatment over the surfaces. For contact angle measurements, a typical PIII treatment similar to the above conditions lasting for at least 8 min was given.

### 2.3. Contact Angle Measurements

Wettability is an important measure for biocompatibility as hydrophobic surfaces may denature soluble host proteins, which rely on a hydrophobic core to maintain their functional confirmation, by hydrophobic interaction of the surface with their core. Contact angle measurements were performed by sessile drop technique applied to a 1 or 4 µL water droplet on the surfaces (*n* = 6 or more). The measurements were taken by a droplet contact angle goniometer (Kruss, Hamburg, Germany) according to the manufacturer’s instructions or by photographic assessment.

### 2.4. Peptide Immobilisation

Active peptide Mel4 (amino acid sequence KNKRKRRRRRRGGRRRR) and inactive peptide Mel1 (amino acid sequence TLISWIQRPRVS) [29] were sourced from Auspep (Melbourne, VIC, Australia). Peptide immobilisation was carried out by overnight incubation at room temperature of untreated and PIII-treated PAEK specimens (9 at a time) in 1 mL of a 2 mg/mL peptide in phosphate-buffered saline (PBS) or PBS only in a standard 1.5 mL polypropylene microcentrifuge tube under mild agitation. Subsequently, the discs were washed 3 times with PBS to remove excess peptides followed by 3 washes with distilled water. Then, the specimens were airdried and sterilised by steam autoclaving. This immobilisation method was chosen under the assumption that the peptide is in excess for a monolayer coverage over the specimen surface. All specimens have been stored at room temperature until further use.

### 2.5. Antimicrobial Studies

These studies used a modified version of an adhesion assay that had been previously used to assess the ability of covalently bound Mel4 to prevent adhesion of *S. aureus* to contact lenses, which were then used in Phase II/III clinical trials to measure their ability to control bacterially driven adverse events during contact lens wear [28,34]. 

An overnight culture of *S. aureus* (SA 38) was diluted in Mueller–Hinton broth media to a final concentration of 10^5^ cells/mL. The bacterial suspension was then added to either PIII-treated or untreated discs that either had no peptide, Mel1 or Mel4 immobilised to them. The discs were then incubated in a humidified chamber for 24 h at 37 °C. After incubation, the media was discarded and the surfaces of the discs were washed thoroughly with PBS. After the final wash, the discs were submerged in PBS and vortexed vigorously to resuspend the strongly attached bacteria in the PBS. Serial logarithmic dilutions of the obtained PBS solution were prepared and subsequently plated on agar plates. After 24 h of incubation, the number of bacterial colonies growing on them was counted. The number of colony-forming units (CFU) per mL of the undiluted PBS solution was determined by multiplying the results with appropriate dilution factors. 

### 2.6. Cell Culture

The osteosarcoma cell line Saos-2 was used here as a model for bone–implant interactions. The cell line is known for its ability to differentiate from an early osteoblast-like cell to a late osteoblast-like cell when a preferable substrate is present. Saos-2 cells were purchased from the American Type Culture Collection. The basal growth media consisted of McCoy’s 5A medium (Gibco/Life Technologies Australia Pty. Ltd., Mulgrave, Australia) supplemented with 15% foetal bovine serum (FBS; Gibco/Life Technologies Australia Pty. Ltd., Mulgrave, Australia). The cells were cultured under standard culture conditions at 37 °C in a humidified atmosphere with 5% CO_2_.

### 2.7. Cell Adhesion

Adhesion of Saos-2 cell in vitro is a useful indicator for the performance of a biomaterial in vivo. Saos-2 cell adhesion was assessed by AlamarBlue (AB; Invitrogen/Life Technologies Australia Pty. Ltd., Mulgrave, Australia) assay conducted according to the manufacturer’s instructions. AB is a resazurin-based cell substrate; its fluorescent metabolite is used as a measure of cell viability and from that of cell number. For the cell adhesion test, Saos-2 cells were seeded onto wetted PAEK specimens containing either immobilised Mel4 or no peptide. The cells were seeded at a density of 1 × 10^5^ cells/cm^2^ in 96-well plates in serum-free cell culture medium. Serum-free medium was chosen to ensure the effect of Mel4 is not obstructed by other proteins and peptides in solution. The cells were allowed to adhere to the specimen for 3 h before careful washing with FBS-containing media to remove unbound and loosely attached cells. Then, the cells were cultured for a further 48 h to allow them to fully adhere to the specimens. On the day of the AB assay, the PAEK discs with the attached cells were transferred into wells of new 96-well plates and incubated with 120 µL of 10% AB in culture medium and incubated for 2 h. The fluorescent signal of 100 µL was measured with a CLARIOstar microplate reader (BMG LABTECH Pty. Ltd., Mornington, Australia) at an excitation wavelength of 545–20 nm, emission wavelengths of 600–40 nm (gain set at 1522).

### 2.8. Proliferation and Mineralisation

An assay for the assessment of biocompatibility of biomaterials is the degree of cellular and acellular mineralisation. Surface modification can lead to the inhibition of acellular mineral deposition and nucleation. It can also cause cells contacting the material to produce fewer factors important for biomineralisation. Mineral deposits can be stained with Alizarin Red S (ARS), a nonspecific calcium dye, and quantified by subsequent elution under acidic conditions. The absorbance of the eluent is proportional to the amount of calcium in the sample. The effect of immobilised Mel4 and the effect of plasma treatment status on Saos-2 cell-mediated calcium deposition was determined. Saos-2 cells were seeded onto the specimen at a density of 1 × 10^4^ cell/cm^2^ and incubated for 35 days in culture under standard culture conditions with biweekly growth medium changes. The initial proliferation was monitored by AB assay as described above (gain set at 1110). On the final day, the specimens were moved to a new culture vessel and a PrestoBlue (PB, Invitrogen/Life Technologies Australia Pty. Ltd., Mulgrave, Australia) assay was conducted according to the manufacturer’s instructions to measure the viability of the final cell population. The cell number on each sample was estimated based on a PB calibration curve with known cell numbers. Then, the specimens were fixed in 4% paraformaldehyde in PBS for 20 min. For the ARS procedure, the cells were washed briefly with distilled water before adding a 2% ARS/H_2_O solution (Sigma-Aldrich/Merck Life Science Pty. Ltd., Bayswater, Australia) to the cells. After 15 min, the ARS solution was removed, and the specimens were washed several times with distilled water until the water ran clear. ARS dye was eluted with 200 µL of a 10% acetic acid, 10% methanol water solution for 45 min under gentle orbital shaking. The absorption at 425 nm of 180 µL of the eluent was measured with a CLARIOstar microplate reader. Each total ARS signal was normalised to the cell number of the respective specimen. 

### 2.9. Cytotoxicity of Unbound Mel4

Cytotoxicity of short-term exposure with free Mel4 was assessed by AB assay. In brief, Saos-2 cells were seeded at a density of 8 × 10^4^ cells/cm^2^ into culture vessels. On the following day, growth medium was replaced with media containing Mel4 in graded concentrations up to 3 mg/mL Mel4 peptide in culture medium and incubated for either 24 h or 72 h. Then, the culture medium was removed, and an AB assay performed as described in Section 2.7. 

### 2.10. Scanning Electron Microscopy 

Visualisation of the bacterial cell and bone–cell interaction with PAEK substrates gives an insight into the mode of attachment and cannot be adequately determined by light microscopy due to opacity of the PAEK polymers. Electron microscopy offers a good solution to this issue but also enables the composition of any phosphate mineralisation to be determined by energy dispersive spectroscopy (EDS). For SEM preparation, the specimens containing cells were fixed with 2.5% glutaraldehyde and dehydrated in graded ethanol before critical point drying of the samples in either HMDS (used for the bone cells) or a critical point dryer (used for bacterial cell analysis). Surfaces of the bone cell-containing samples were imaged with a PhenomXL (Thermo Fisher Scientific, Waltham, MA, USA) using a backscattered electron detector and an incident electron voltage of 5 kV and air pressure of 60 mPa. The bacterial cells specimens were sputter-coated with 30 nm of platinum before imaging with an FEI Nova NanoSEM 230 (Thermo Fisher Scientific, Waltham, MA, USA). A secondary electron detector and an accelerating voltage of 5 kV and a spot size of 3 nm were used for imaging. 

### 2.11. Statistical Analysis

Statistical analysis was conducted using GraphPad Prism Version 10.0.0. For tests between multiple groups, *p*-values were computed with an uncorrected Fisher’s least significant difference test following a one-way ordinary analysis of variance (ANOVA) or else one-tailed *t*-tests were used.

## 3. Results 

### 3.1. Surface Characterisation

**Figure 1:**
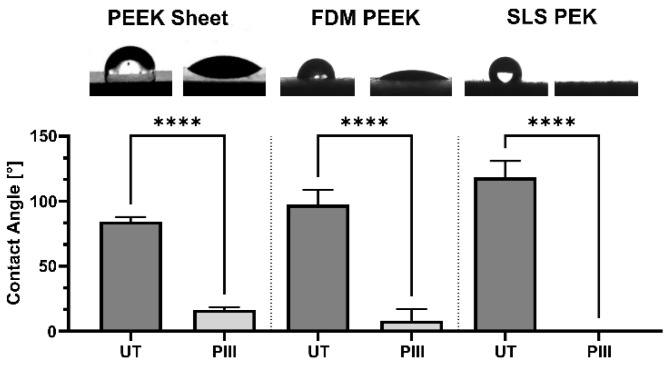
Water contact angles of untreated (UT) and plasma immersion ion implantation (PIII)-treated PEEK sheet, FDM-printed PEEK, and SLS-printed PEK surfaces, arranged in order of increasing roughness. Shown are mean values and standard deviation (*n* ≤ 6). The measurement for PIII-treated SLS PEK is 0°. Above the graph are photographic images of a 4 µL (PEEK sheet) or a 1 µL (FDM PEEK and SLS PEK) water droplet on the respective surfaces. **** *p* ≤ 0.0001.

Contact angle reduction in PEEK sheet, FDM PEEK, and SLS PEK was observed after a standard PIII treatment (Figure 1). Water contact angles behave according to the Wenzel equation and increase with roughness in the case of untreated, more hydrophobic surfaces and decrease with roughness for PIII-treated, hydrophilic surfaces at the roughness scale of our measurement [39]. 

### 3.2. Antimicrobial Effect of Mel4

**Figure 2:**
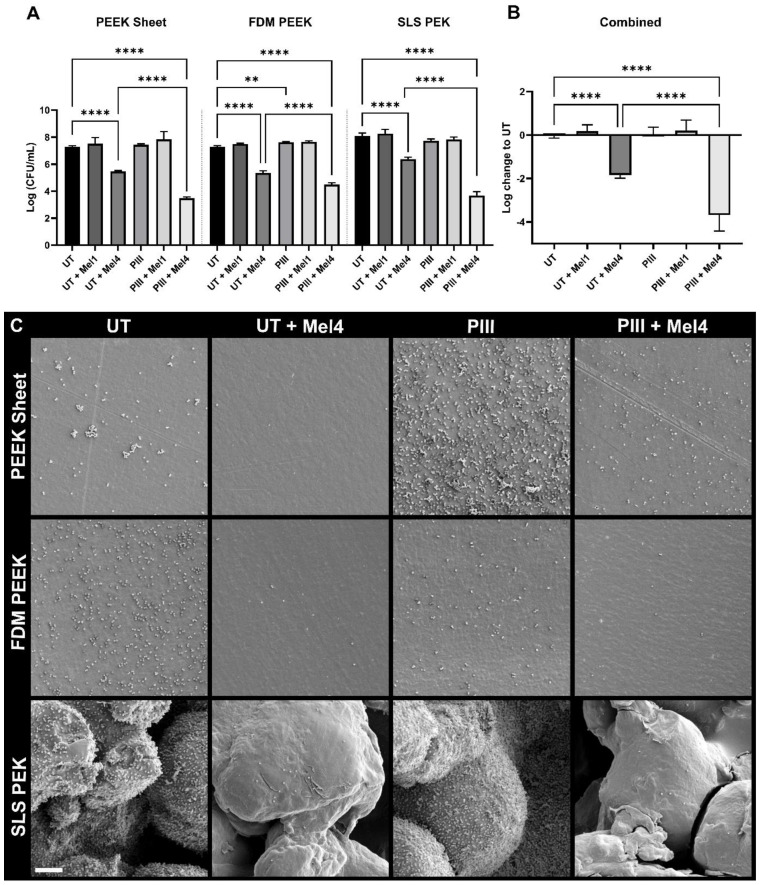
Effectiveness of Mel4 bound to PAEK specimens against *S. aureus* using inactive peptide Mel1 as control. (**A**) The microbial counts in logarithms of the colony-forming units (CFUs)/mL on PEEK sheet, FDM PEEK, and SLS PEK surfaces with respect to untreated (UT) and plasma immersion ion implantation (PIII)-treated surfaces with either no peptide, Mel1 or Mel4 immobilised to them. Shown are the mean values and standard deviation of 3 replicates. (**B**) All available data from graph A combined, equally weighted, normalised to UT and graphed irrespective of the substrate material. Mel4 shows a 1.8 log reduction on the UT surface and a 3.7 log reduction on PIII-treated surfaces. Mel1 does not significantly increase or decrease bacterial adhesion on UT or PIII-treated surfaces. Some significance bars are omitted for readability. ** *p* ≤ 0.01; **** *p* ≤ 0.0001 (**C**) Mode of *S. aureus* adhesion to PAEK specimens. Note: SEM micrographs are not quantitatively interpretable. Scale bar = 10 µm.

Figure 2 shows the results for the effectiveness of Mel4 against *S. aureus* on all surfaces. Overall, Mel4 gave a bacterial count reduction of 1.8 log on untreated surfaces and 3.7 log on PIII treated. The control inactive peptide Mel1 did not change the microbial count significantly. The PEK material led to an overall increase in bacterial adhesion compared to the PEEK materials when no Mel4 was present (*p* ≤ 0.05, not graphed). This was probably due to higher roughness that can trap bacteria solution during the initial adhesion phase of the assay and the increased surface area of the PEK specimens. Importantly, the greater surface roughness and area has not impeded the antimicrobial action of the Mel4, which has reduced the microbial count to the same level as for the smoother PEEK surfaces.

SEM studies revealed the coverage of bacterial cells on specimens not containing Mel4 appears uniform (Figure 2C). Bacterial cells do not seem to prefer to occupy textured areas on the 3D-printed specimen but are distributed uniformly. However, on SLS-printed surfaces, which have a larger surface porosity, there are more cells overall, distributed over the increased surface area. 

### 3.3. Cytotoxicity of Mel4

**Figure 3:**
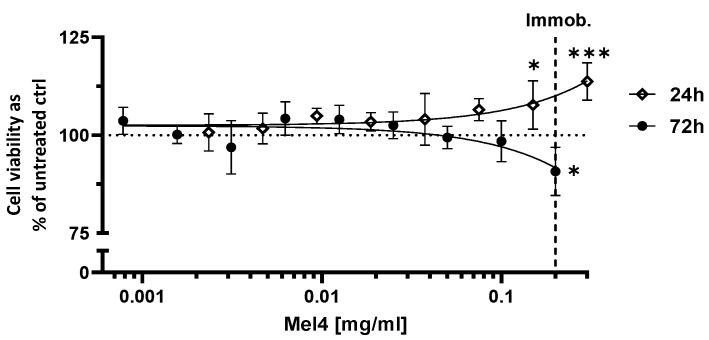
Viability of Saos-2 cells cultured for 24 and 72 h with free Mel4 in solution as a function of the concentration. Each point represents the mean value and the standard deviation of 3 replicates. The lines correspond to linear regression curves. Immob.: Concentration used to immobilise Mel4 to PAEK specimens. * *p* ≤ 0.05; *** *p* ≤ 0.001.

The possibility of a cytotoxic effect of free Mel4 on Saos-2 cells was investigated. Unbound Mel4 at a concentration of 2 mg/mL had a very slight cytotoxic effect on Saos-2 after 72 h of incubation as seen by the reduction in cell viability by 9% compared to untreated cells. On the other hand, short-term exposure to free Mel4 at high concentrations of 0.15 and 0.3 mg/mL shows growth-enhancing effects of up to 13%. The concentration of 2 mg/mL corresponds to the peptide concentration during immobilisation to PAEK surfaces. At concentrations lower than 0.15 mg/mL, there was no detectable effect on the viability on Saos-2 cells after either 24 h or 72 h (Figure 3). 

### 3.4. Bone Cell Adhesion

**Figure 4:**
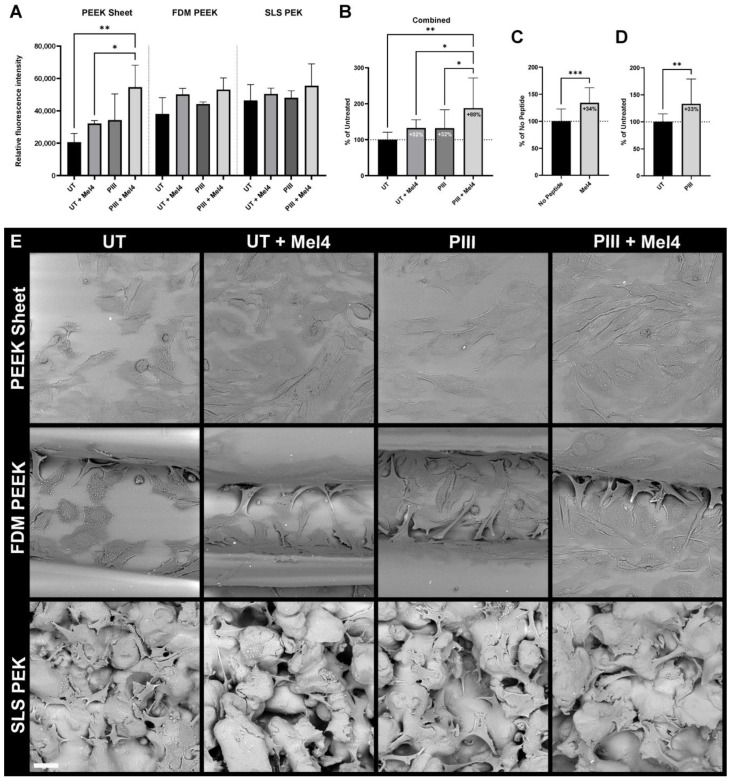
Saos-2 cell adhesion to PAEK surfaces. (**A**) Cell adhesion to PEEK sheet, FDM PEEK, and SLS PEK surfaces either untreated (UT) or plasma immersion ion implantation (PIII)-treated with either no peptide, or Mel4 immobilised to them. The mean value and standard deviation of 2–3 replicates are shown. (**B**) Effect of PIII and Mel4 on cell adhesion, irrespective of substrate material, obtained by combining data from A, equally weighted and normalised to UT. (**C**) Effect of presence of Mel4 obtained by combining data from A, irrespective of substrate material and treatment status, equally weighted and normalised to no peptide. (**D**) Overall effect of PIII treatment obtained by combining data from (**A**), irrespective of substrate material and presence of peptide, equally weighted and normalised to UT. * *p* ≤ 0.05; ** *p* ≤ 0.01; *** *p* ≤ 0.001. (**E**) SEM micrographs of Soas-2 cells adhered to PAEK specimens after 48 h of incubation. Scale bar = 30 µm.

Adhesion of Soas-2 cells to PAEK surfaces was increased with the presence of Mel4 and with PIII treatment on all polymeric substrates (Figure 4). All available data for all surface types combined, equally weighted, and normalised to untreated demonstrate an increase in cell adhesion by 32% with the presence of Mel4 and an increase by 32% with PIII treatment. Covalently linked Mel4 on the PIII-treated surfaces increases cell adhesion by 88%.

### 3.5. Proliferation and Mineralisation

**Figure 5:**
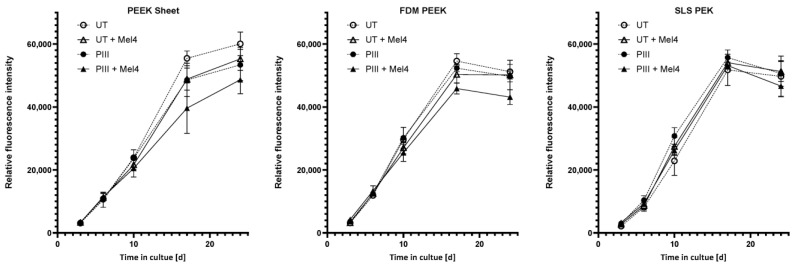
Initial cell proliferation of Saos-2 cells on PAEK surfaces. Results are shown for PEEK sheet, FDM PEEK, and SLS PEK surfaces either untreated (UT) or plasma immersion ion implantation (PIII)-treated with either no peptide or Mel4 immobilised to them. Each point represents the mean value and the standard deviation of 3 replicates. There was no significant reduction in the growth at each time point.

We investigated the effect of Mel4 on proliferation and mineralisation capabilities of Saos-2 cells on PAEK by culturing cells on the polymeric specimens for a total of 35 days. Figure 5 shows the initial cell proliferation as monitored over 24 days via AB assay.

**Figure 6:**
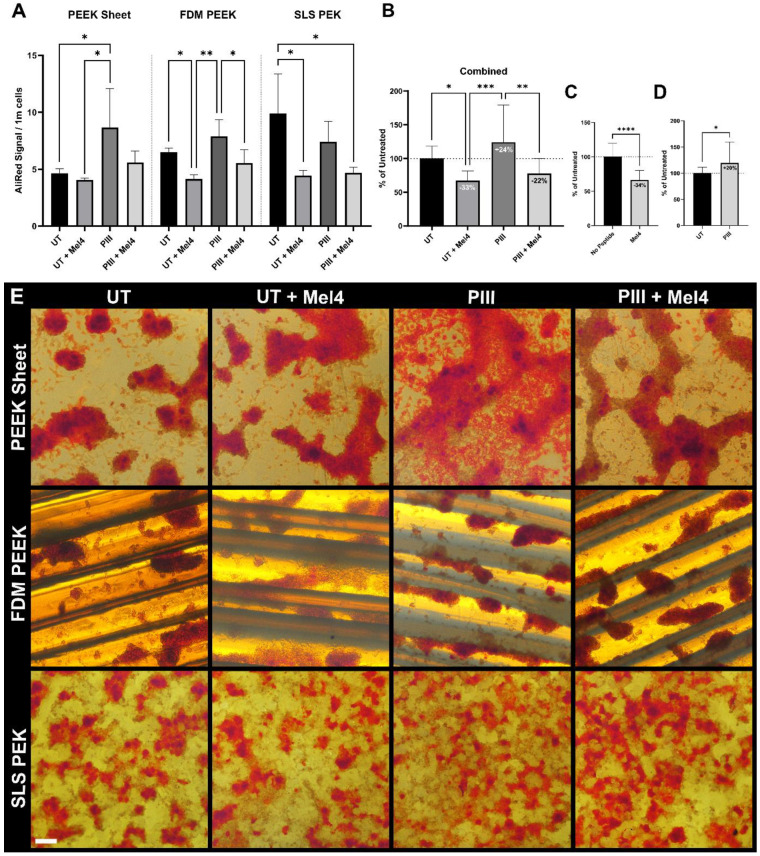
Saos-2 cell mineralisation on PAEK surfaces. (**A**) Mineralisation per million Saos-2 cells assessed by PrestoBlue and Alizarin Red S assays. Results are shown for PEEK sheet, FDM PEEK, and SLS PEK surfaces either untreated (UT) or plasma immersion ion implantation (PIII)-treated with either no peptide or Mel4 immobilised to them. The mean value and standard deviation of 2–3 replicates are shown. (**B**) Effect of PIII and Mel4 on mineralisation, irrespective of substrate material, obtained by combining data from A, equally weighted and normalised to UT. (**C**) Effect of presence of Mel4 obtained by combining data from A, irrespective of substrate material and treatment status, equally weighted and normalised to no peptide. (**D**) Overall effect of PIII treatment obtained by combining data from (**A**), irrespective of substrate material and presence of peptide, equally weighted and normalised to UT.* *p* ≤ 0.05; ** *p* ≤ 0.01; *** *p* ≤ 0.001. **** *p* < 0.0001. (**E**) Light microscopic images of Alizarin Red S staining. 40× magnification. Scale bar = 0.5 mm.

The presence of mineral was assayed at the end of the experiment. Figure 6 shows the mineralisation signal per one million cells to account for the different surface areas produced by different values of roughness. Overall, Mel4 reduced mineral detection by 34%; PIII increases it by 20% (Figure 6C,D). 

**Figure 7:**
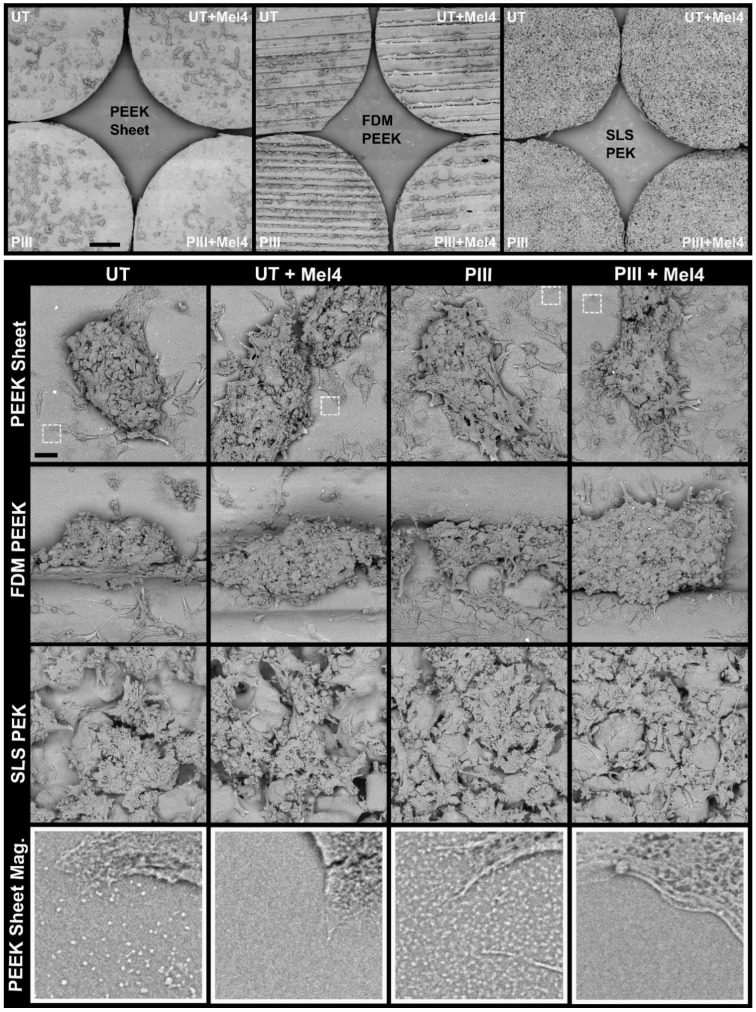
Morphology of Saos-2 cells after 35 days in culture on PAEK specimens and after performance of PrestoBlue and Alizarin Red S assays. SEM images reveal cellular and acellular contributions to mineralisation. Top panel: Overview SEM micrographs of Saos-2 growth on PAEK specimens. Saos-2 cells formed clusters, typical for late-stage differentiation. On rougher FDM PEEK and PEK specimens, the clusters occupy valleys created by the 3D process within the surface. Scale bar = 1 mm. Lower panels: Interaction of cells with the respective surface. PIII-treated samples show better coverage of individual cells on the surface independent of the presence of Mel4. Bottom panel: Magnified areas from PEEK sheet micrographs (white dotted boxes). Close-up images reveal surface features on specimen not containing Mel4. Scale bar = 30 µm. UT: Untreated, PIII: Plasma Immersion Ion Implantation.

Light microscopic imaging of the cells during the ARS staining procedure (Figure 6E) and SEM imaging after elution of the stain (Figure 7) revealed Saos-2 cells formed cell clusters typical for late-stage mineralisation on all substrate types. On FDM-printed surface, the cell clusters predominantly occupied the valleys created by the 3D printing nozzle. On SLS PEK surfaces, the cells grew within the surface porosity. 

Areas on PEEK sheet and FDM PEEK samples without Mel4 that were not covered with Saos-2 cell showed small deposits that potentially contained mineral before elution. Determination of the presence of the features on the PEK specimen was inconclusive due to the inherent small-scale roughness of PEK. However, the depositions were also found, although to a much lesser degree of coverage, on Mel4-free PEEK specimens that were not incubated with cells but with culture media containing foetal bovine serum.

## 4. Discussion

The significant differences of the effect of Mel4 attachment and PIII treatment, summarised in Table 3, can be attributed to the different modes of attachment and the resulting change of the orientation of the peptide to untreated and PIII-treated polymer surfaces. 

**Figure 8:**
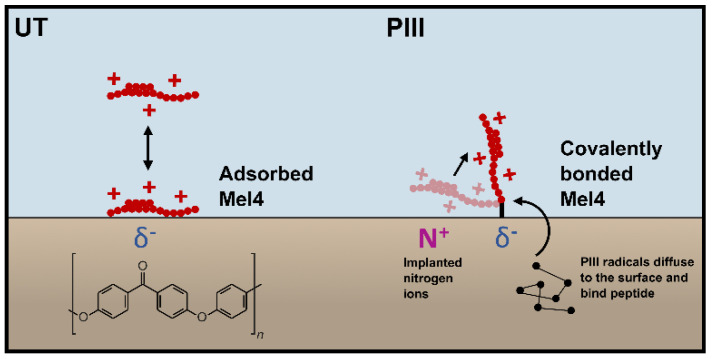
Illustrating the different modes of attachment of Mel4 to untreated (UT) and PIII-treated surfaces (using the example of a PEEK surface). A partial negative charge (δ^−^) on the untreated surface adsorbs Mel4 by attracting its positive charge (+), orienting it along the surface. Meanwhile, PIII-generated radicals buried in the subsurface diffuse to the surface and bind the peptide from solution. The positively charged nitrogen ions, implanted during PIII treatment, give the surface overall a more positive charge that may help to erect the peptide into an orthogonal orientation, more favourable for antimicrobial action.

Mel4 is highly positively charged, with 14 out of 17 amino acid residues carrying a positive charge at physiological pH, giving it a theoretical isoelectric point of 13.5. Mel4 has no hydrophobic side chains and is predicted to form an alpha helix flanked by two short coils of two and seven amino acids. The cationic character and the alpha helix conformation is believed to facilitate the antimicrobial action of Mel4 by interaction with the phosphate groups of lipid A and by penetrating the cell membrane of the bacterium and causing membrane depolarisation [7]. PEEK and PEK have borderline hydrophobic surfaces and with added surface roughness the water contact angle increases to hydrophobic. Mel4 near an untreated surface is potentially adsorbed by the means of partial negatively charged oxygen atoms in PEEK and PEK (Figure 8). The peptide would then orient along the plane of the surface, giving it less opportunity to penetrate the bacterial membranes. Adsorbed Mel4 on the untreated surface is likely to desorb and be washed away by a change of media and/or be replaced by other larger molecules due to the Vroman effect. Both effects lead to an overall dilution of the peptide on the surface over time. PIII treatment facilitates a very different mode of attachment by introducing long-lived radicals that can travel along the polymer chains and covalently bind predominantly carbon-containing molecules at the surface [11]. Mel4 being covalently attached to PEEK and PEK may then orient itself orthogonal to the surface due to the repulsion by the implanted positively charged nitrogen ions (Figure 8). The surface becomes more positively charged overall, potentially facilitating the adhesion of Saos-2 cell by attraction of the negatively charged plasma membrane. This interaction does not affect proliferation of the cells; hence, there is no reduction in cell growth overall caused by the presence of the peptide. 

Mineralisation, in contrast to proliferation, is negatively impacted by the presence of Mel4. Mineral deposition on cell-free areas on PAEK surfaces is correlated with the presence of small surface features. Although it is possible that the features are calcium phosphate-containing matrix vesicles secreted by Saos-2 cells [40], it is more likely that the features are caused by acellular amorphous calcium phosphate-associated protein depositions whose production is only indirectly mediated by cells [37]. Support for the non-vesicular origin interpretation is that the features were also found on completely cell-free specimens that were incubated in culture media only and that a positively charged Mel4 surface coverage would attract entities such as matrix vesicles with a negative charge. Assuming these features are responsible for the reduced ARS signal of the Mel4 surfaces, the question remains as to whether the cells produce less protein, or the protein they produce cannot adhere to the surface owing to the blockade by Mel4. 

Although we have shown that Mel4 decreases mineralisation in vitro, it is unlikely this action will be sustained in vivo due to host protease degradation of the peptide over time. In silico analysis, developed to predict half-life of peptides in blood based on their sequence, revealed a half-life of only 27 min [41]. However, a phase III clinical trial of covalently attached Mel4 on contact lenses found activity after six days of wear on the cornea [42]. PIII and its effect on bioactivity is likely to extend well beyond the lifetime of the peptide in vivo.

## 5. Conclusions

The plasma immersion ion implantation method of creating covalent linkages when used with a broad-spectrum antimicrobial peptide such as Mel4 provides an effective method for reducing the microbial count on PEEK and PEK surfaces while showing no growth-inhibiting effect on osteoblastic cells. The different surface morphologies created by 3D printing processes do not inhibit the efficacy of the antimicrobial. A textured implant surface, such as that of SLS PEK, can be equally well protected as a smooth conventionally produced implant. 

Mineralisation appears to be somewhat negatively affected by the surface coverage of Mel4 in vitro. However, in vivo, it is not anticipated that osseointegration would be adversely affected by Mel4 due to the inherent instability of peptides when exposed to the endogenous proteases of the body. The covalent linking of Mel4 via PIII should bridge the critical time to prevent infection of an orthopaedic implant immediately following the surgical insertion, while the increased bioactivity towards bone regeneration introduced by PIII will outlast that period. This should prompt successful osseointegration of the implant to provide long-term protection against infection. An in vivo trial is strongly recommended as the next step.

## Figures and Tables

**Table 1:** FDM PEEK 3D printing parameters.

Parameter	Value
Nozzle temperature	440 °C
Print bed temperature	200 °C
Chamber temperature	120 °C
Layer height	0.2 mm
Nozzle diameter	0.2 mm
Extrusion width	0.25 mm
Print speed	5 mm/s

**Table 2:** SLS PEK 3D printing parameters.

Parameter	Value
Layer thickness	0.12 mm
Build area configuration	230 mm × 350 mm
Process chamber temperature	364 °C
Building platform temperature	336 °C
Exchangeable frame temperature	343 °C
Post sintering time	12 s
Beam offset	0.41 mm
Laser exposure set	PAEK1304_120_011
Cooldown	Passive ambient

**Table 3:** Summary of main results. 0 = no change, + + = strong increase, + = increase, − − = strong decrease compared to UT.

	UT	UT + Mel4	PIII	PIII + Mel4
Protection from bacterial cell adhesion	0	+	0	+ +
Bone cell adhesion	0	+	+	+ +
Bone cell proliferation	0	0	0	0
Bone cell-mediated mineralisation	0	− −	+	0

## Data Availability

Data are contained within the article.
